# A research framework for evaluating upstream multimodal sarcopenia risk domains in older adults

**DOI:** 10.3389/fmed.2026.1928432

**Published:** 2026-10-14

**Authors:** Nackhwan Kim

**Affiliations:** Institute of Human Behavior and Genetics, Korea University College of Medicine, Seoul, Republic of Korea

**Keywords:** biomarkers, early detection, muscle composition, narrative review, neuromuscular aging, older adults, risk domains, sarcopenia

## Abstract

Established sarcopenia definitions identify clinically important impairment, but their thresholds are not designed to characterize all biological changes associated with subsequent muscle decline. Whether additional measurements improve assessment before these thresholds are reached remains uncertain. This structured narrative review proposes a research framework for evaluating upstream, multimodal sarcopenia risk domains in older adults. It integrates diagnostic consensus statements with mechanistic, observational, diagnostic-accuracy, prognostic, and intervention literature identified through purposive searches and citation tracking. Candidate domains comprise circulating biomarkers, creatine-pool-based muscle mass estimation, neuromuscular measurements, imaging and bioelectrical measures, and digital mobility assessments. The evidence supporting these domains is heterogeneous. Cross-sectional differences and biological plausibility do not establish prediction of incident sarcopenia, incremental clinical value, or differential treatment benefit. Neuromuscular abnormalities may reflect aging rather than sarcopenia specifically, and measures commonly grouped as muscle quality assess distinct constructs. The proposed domains are therefore nonexclusive and nonsequential; no diagnostic stages, validated multimodal score, or biomarker-guided treatment algorithm are proposed. Conventional clinical assessments provide reference phenotypes, while falls, disability, fractures, quality of life, hospitalization, and mortality remain separate validation outcomes. The principal contribution is an evidence-to-validation framework that distinguishes demonstrated measurement capabilities from untested applications. Priorities are longitudinal assessment, reproducibility, external validation, incremental prediction beyond inexpensive clinical measures, and trials of clinical utility before implementation.

## 1 Introduction

The term sarcopenia was introduced to describe age-associated loss of skeletal muscle, and early operational definitions emphasized appendicular lean mass relative to body size (1, 2). Subsequent evidence linked strength and physical performance more consistently than lean mass alone to important adverse outcomes. This shifted diagnostic attention toward muscle function as well as quantity (3–6).

Current diagnostic thresholds are essential clinical anchors, but they are not primarily designed to detect the earliest biological alterations associated with muscle decline (6–8). Neuromuscular remodeling, altered tissue composition, and metabolic or inflammatory disturbances may be detectable before an individual meets a conventional definition. However, detection of an abnormality before a threshold is reached does not establish that it predicts future sarcopenia or that acting on it improves outcomes (9–12).

The possibility of earlier assessment is relevant to prevention, but intervention should not be delayed pending investigational testing. Exercise, nutritional assessment, and management of reversible contributors already have a place in appropriate older populations (13–15). Sarcopenia may be at least partly reversible; neither an inevitable progression nor an irreversible functional endpoint is assumed here (16). The Asian Working Group for Sarcopenia (AWGS) 2025 life-course muscle-health approach already extends prevention beyond established sarcopenia (17).

The principal contribution of this review is an author-proposed research framework for evaluating upstream, multimodal sarcopenia risk domains. It organizes what candidate measurements assess, what study designs support them, and what validation is still required (18–20). It is not a new staging system, a derived prediction model, or a clinical treatment algorithm. Its intended clinical focus is older adults, generally aged 65 years or above; evidence from other age groups is retained when relevant to mechanisms or life-course context, rather than treated as directly generalizable.

In this review, upstream means assessment before a selected conventional diagnostic threshold, not a proven earlier point in a universal disease sequence. Early detection means identifying a candidate abnormality before that threshold; screening means testing to identify people who require further assessment. Risk stratification assigns future risk, phenotyping describes current characteristics, and prediction estimates a specified future outcome over a stated time horizon. Response monitoring measures change after an intervention; it does not by itself predict which treatment provides greater benefit. The term candidate denotes a proposed use requiring validation, not an established ability to stratify future risk.

## 2 Review approach and evidence synthesis

This is a structured narrative review, not a systematic review or meta-analysis. Its purpose is to integrate heterogeneous clinical and mechanistic literature and develop questions for validation, rather than estimate a pooled effect. Principles of transparent narrative synthesis informed the approach (21). Literature identification and interpretation were undertaken by the single author; there was no duplicate independent screening or formal risk-of-bias assessment conducted for this review.

Literature identification used purposive searches in PubMed/MEDLINE, Embase, Web of Science Core Collection, Scopus, and the Cochrane Library, supplemented by backward and forward citation tracking. The original purposive literature-identification searches were last conducted in May 2026. Subsequent revisions involved targeted source verification and additions rather than a comprehensive update across all literature-identification domains. The searches were iterative and intended to identify informative sources, not to provide an exhaustive, reproducible systematic search. Database-specific Boolean strategies and a prospectively recorded screening flow are not provided. Historical retrieval and exclusion counts cannot be reconstructed from the cited bibliography and are therefore not estimated.

Supplementary Table S1, entitled “Purposive evidence-identification and narrative-selection framework,” presents nine literature-identification domains coded D1–D9. These organize source selection and are distinct from the five candidate measurement families evaluated in this review. The table describes representative concepts, narrative-selection priorities, conceptual scope and interpretation limits, and each domain's role in the review. It is not a set of retrospectively reconstructed search strings or prespecified eligibility criteria. Consensus statements were selected for their influence on sarcopenia definitions. Primary human studies were emphasized for design-specific claims about measurement, classification, prognosis, and intervention. Systematic reviews were used to characterize the breadth and limitations of a domain, while narrative reviews provided context and routes to primary evidence. Preclinical evidence was retained for mechanisms and explicitly separated from human temporal or diagnostic evidence.

When several sources addressed the same measurement, priority was given to direct relevance to older adults, a clearly specified construct and reference definition, longitudinal design for prognostic claims, and primary evidence for technical claims. Recency alone did not determine priority. Null or discordant findings were retained, and differences in population, assay, diagnostic definition, and design were used to interpret disagreement. Multiple reports or reviews of overlapping cohorts were not regarded as independent replications. Technologies without a relevant muscle or functional construct were outside the conceptual scope; lower narrative priority was not treated as a formal exclusion decision. Full-text interpretation was limited to English-language reports, with English abstracts used for citation discovery.

The bibliography of the initial submission contained 71 distinct source records. Four sources were added during the first revision to clarify motor-unit indices, sarcopenic obesity, frailty assessment, and longitudinal C-terminal agrin fragment evidence, resulting in 75 mapped cited sources. The source-level map retains 75 cited sources, with design categories and nonexclusive domain assignments in Supplementary Table S2. Supplementary Table S1 reports the corresponding reference numbers and counts for D1–D9. Each distinct cited source is counted once within every domain to which it is mapped; the 75 sources produce 112 source–domain assignments. These are descriptive counts of the cited evidence base, not database search yield, screened records, independent participant cohorts, or evidence-certainty scores. No cited sources or source–domain assignments were added or removed in the present consistency revision. Targeted verification during revision does not constitute a new comprehensive search update.

Evidence was distinguished as mechanistic, cross-sectional, diagnostic-accuracy, longitudinal/prognostic, interventional, or methodological/consensus evidence. Monitoring responsiveness, prediction of treatment benefit, and improved clinical decisions were assessed as separate claims. In Supplementary Table S1, D8 addresses intervention efficacy in the populations studied and measurement responsiveness; predictive enrichment remains a future research question requiring separate evidence of treatment-effect modification. Tables 3, 4 provides descriptive evidence and readiness categories; these are not formal GRADE ratings. Where a cited systematic review reported risk of bias or certainty, its own assessment is identified rather than extrapolated to other domains.

The figures are conceptual syntheses of the cited literature, not additional primary evidence. They were not used to derive diagnostic cutoffs, stage boundaries, or a prediction score.

## 3 Diagnostic frameworks and the added value of domain-based research

The European Working Group on Sarcopenia in Older People (EWGSOP) 2010 consensus combined low muscle mass with low strength or performance and used presarcopenia for low mass without accompanying strength or performance impairment (3). EWGSOP2 identifies probable sarcopenia from low strength, confirms it through low muscle quantity or quality, and uses physical performance to indicate severity (6). AWGS 2019 introduced possible sarcopenia to facilitate community intervention when low strength or reduced performance is present (7). These categories already support action before severe disease and should not be portrayed as waiting for late disability.

The Sarcopenia Definitions and Outcomes Consortium (SDOC) emphasizes low grip strength and slow usual gait speed because of their outcome associations (22). The Global Leadership Initiative in Sarcopenia (GLIS) accepts muscle mass, strength, and muscle-specific strength as conceptual components, while treating physical performance as an outcome. Its Delphi process did not reach the prespecified acceptance threshold for disease-severity levels (16). Consequently, the present review does not infer a consensus-endorsed stage sequence from GLIS.

AWGS 2025 explicitly adopts life-course muscle health, extends assessment to adults aged 50–64 years, requires concurrent low muscle mass and strength for diagnosis, and treats physical performance as an outcome measure (17). The additional contribution proposed here is narrower: mapping investigational domains to the evidence needed for prediction and clinical utility. It does not claim novelty for early prevention, life-course assessment, or the separation of components and outcomes.

Korean Working Group on Sarcopenia (KWGS) functional sarcopenia recognizes low strength together with low performance despite preserved measured muscle mass (23, 24). Dynapenia describes age-related strength loss and is not interchangeable with low muscle mass (25). Multidomain frailty assessment addresses broader vulnerability, including health and functional deficits beyond skeletal muscle (26). In contrast, the present domain framework asks how candidate muscle-related measurements should be tested alongside, rather than substituted for, these established clinical phenotypes. Definitions identify partially overlapping populations; prevalence, agreement, and outcome associations differ accordingly (27–31). Table 1 summarizes these distinctions.

**Table 1:** Established concepts and the distinct purpose of upstream domain research

Framework or concept	Existing clinical/conceptual role	Relation to earlier assessment	What this review adds—not replaces
EWGSOP 2010/presarcopenia (3)	Low mass with low strength or performance; presarcopenia denotes isolated low mass.	Already recognizes a mass-only phenotype.	Questions whether other measurements predict subsequent impairment; no new mass-only category.
EWGSOP2 (6)	Low strength: probable; low quantity/quality: confirmation; low performance: severity.	Probable sarcopenia permits action before confirmation or severe disability.	Investigational domains are assessed alongside these clinical anchors.
AWGS 2019 (7)	Possible sarcopenia: low strength or performance in community assessment.	Encourages early conventional exercise/nutritional care.	Does not rename possible sarcopenia as a novel biological stage.
AWGS 2025 (17)	Life-course muscle health; diagnosis requires low mass and strength; performance is an outcome.	Extends assessment to ages 50–64; prevention precedes established disease.	Maps evidence and validation needs; does not claim novelty for life-course prevention.
GLIS (16)	Conceptual components: mass, strength, muscle-specific strength; potential reversibility.	Separates components from outcomes; severity levels did not meet the acceptance threshold.	No consensus-endorsed sequential states or interchangeable quality measures are inferred.
SDOC (22)	Outcome-oriented low grip strength and slow usual gait speed.	Identifies clinically relevant outcome-risk phenotypes.	Tests added value rather than agreement with a single reference definition.
KWGS/functional sarcopenia (23, 24)	Low strength together with low performance despite preserved measured mass.	Recognizes functional risk not captured by low-mass criteria alone.	Keeps the clinical phenotype distinct from experimental biological measurements.
Dynapenia (25)	Age-related loss of strength, distinct from low muscle mass.	Strength loss need not track lean-mass loss.	Examines possible correlates/predictors without relabeling dynapenia.
Multidomain frailty (26)	Broader health and functional vulnerability beyond skeletal muscle.	Supports whole-person geriatric assessment.	Focuses on muscle-related measurement questions, not a replacement frailty scale.

Frameworks identify overlapping but nonidentical phenotypes. The proposed research domains have no validated entry thresholds, diagnostic cutoffs, or obligatory order.

## 4 Clinical anchors, population context, and measurement constructs

Grip strength, gait speed, chair stands, and the Short Physical Performance Battery (SPPB) provide inexpensive and prognostically relevant clinical information (6, 8, 32, 33). Strength can decline faster than measured lean mass, but this does not establish a single preceding biological pathway (4, 5, 9, 10). Physical performance integrates muscle function with balance, sensation, cognition, cardiopulmonary reserve, pain, psychological status, and environmental demands (34–36). These influences make performance clinically valuable while limiting its specificity for muscle pathology.

Age-related sarcopenia should be distinguished from secondary muscle loss associated with malignancy, neurological disease, organ failure, inflammatory illness, malnutrition, hospitalization, and disuse. These contributors can coexist, and disease-specific findings require explicit qualification when applied to generally healthy older adults (6, 7, 13). Sarcopenic obesity also requires consideration: adiposity may obscure low relative muscle quantity and affect body-size adjustment, impedance estimates, imaging interpretation, and performance. Obesity is not itself proof of sarcopenia (37).

Muscle quality is used as an umbrella term, not as a single interchangeable measurement construct. Computed tomography (CT) attenuation describes radiodensity; magnetic resonance imaging (MRI) fat fraction quantifies a compositional property; ultrasound echo intensity describes image echogenicity; phase angle is a bioelectrical measure; and muscle-specific strength expresses force relative to muscle mass or size. Motor-unit measures characterize neural organization rather than any of these tissue properties (16, 38, 39). Each requires its own measurement model, confounder assessment, and validation endpoint. Table 2 distinguishes the conventional clinical anchors from investigational complements.

**Table 2:** Clinical anchors and distinct measurement constructs.

Measurement	Construct actually assessed	Important limitations/confounders	Role in domain-based research
Grip/chair-stand testing (6, 36)	Strength or integrated task performance, depending on the test.	Effort, pain, cognition, balance and test protocol.	Clinical anchor; candidate measures must add information beyond it.
DXA/BIA estimates (6, 56, 57)	Lean soft tissue or model-estimated muscle mass; not direct contractile protein.	Body size, hydration, population equations and device methods.	Conventional quantity reference; distinguish it from creatine-pool estimation.
Gait speed/TUG/SPPB (32–35)	Integrated physical performance.	Multisystem physiology, cognition, symptoms and environment.	Clinical phenotype and/or outcome, depending on the study design.
D3-creatine dilution (56, 57)	Creatine-pool-based estimate of total-body muscle mass.	Dosing/sampling, conversion assumptions and reference data.	Research measurement; not a measure of force or a validated treatment selector.
CT/MRI (9, 10, 39, 61)	Radiodensity and tissue geometry/fat fraction and composition.	Anatomical site, protocol, contrast conditions, segmentation and body-size adjustment.	Separate compositional predictors; no universal common quality cutoff.
Ultrasound (59, 60)	Regional size, architecture and echogenicity.	Depth, device settings, pressure, operator and image analysis.	Regional measurement with protocol-specific interpretation.
Phase angle (38)	Bioelectrical relation of reactance and resistance.	Fluid distribution, edema, device, nutrition and illness.	Investigational electrical measure; not a defining preclinical stage marker.
Muscle-specific strength (16)	Force relative to a specified mass or size denominator.	Choice of muscle/task, denominator, scaling and measurement error.	Functional ratio, not interchangeable with imaging or bioelectrical composition.
Motor-unit indices (12, 58)	Estimated motor-unit number or related electrophysiological indices.	Method, muscle, contraction and neurological comorbidity.	Neural construct; not direct counts or a tissue-composition measurement.

BIA, bioelectrical impedance analysis; CT, computed tomography; DXA, dual-energy x-ray absorptiometry; MRI, magnetic resonance imaging; SPPB, short physical performance battery; TUG, timed Up and Go.

## 5 Mechanistic rationale and limits of temporal inference

Aging is associated with motor-unit loss, denervation, collateral reinnervation, and enlargement of surviving motor units (40–43). Compensatory remodeling may preserve function, whereas reduced neural activation and impaired muscle contractility can contribute to weakness (44). These observations support neuromuscular investigation, but findings across age or diagnostic groups do not establish individual progression through ordered stages.

Neuromuscular junction changes include structural remodeling, denervation, sprouting, and altered transmission stability (11, 45–47). Animal evidence indicates that some junctional changes can occur before myofiber alterations (45). This provides a mechanistic rationale, not direct proof of the same sequence or a diagnostic window in humans. Inflammatory, endocrine, mitochondrial, regenerative, and tissue-composition disturbances may interact with these changes, occur in parallel, or arise through disease-specific pathways (9, 10, 18, 19).

The human study by Sarto et al. is particularly informative because it also limits disease-specific interpretation (12). Among 130 participants, including 42 younger and 88 older adults, many motor-unit and neuromuscular-junction abnormalities were already present in older adults without sarcopenia. Most did not differ substantially across the older groups. Findings involving near-fiber jitter, C-terminal agrin fragment (CAF), neurofilament light chain, and neural cell adhesion molecule (NCAM)-positive fibers therefore support neuromuscular aging more clearly than discrimination of sarcopenia. The cross-sectional design cannot establish prediction of incident disease.

The electromyographic systematic review identifies motor-unit loss, remodeling, and transmission instability as recurring themes (20). However, its heterogeneous evidence does not validate a universal temporal ordering. Figure 1 provides a conceptual composite of six sarcopenia-related pathophysiologic processes, together with candidate measurement families and established clinical assessment. The continuous tissue background is an illustrative device, not a representation of a single individual, tissue specimen, or longitudinal trajectory. The juxtaposition of features neither requires their coexistence in one individual nor specifies their temporal order; the callout arrows link descriptive labels to illustrative features rather than indicating progression between mechanisms. Myosteatosis/fibrosis and impaired regeneration/remodeling remain distinct, potentially overlapping concepts, and regenerative capacity cannot be inferred from the depicted tissue appearance. Figure 2 presents the corresponding nonsequential research framework and validation questions.

**Figure 1:**
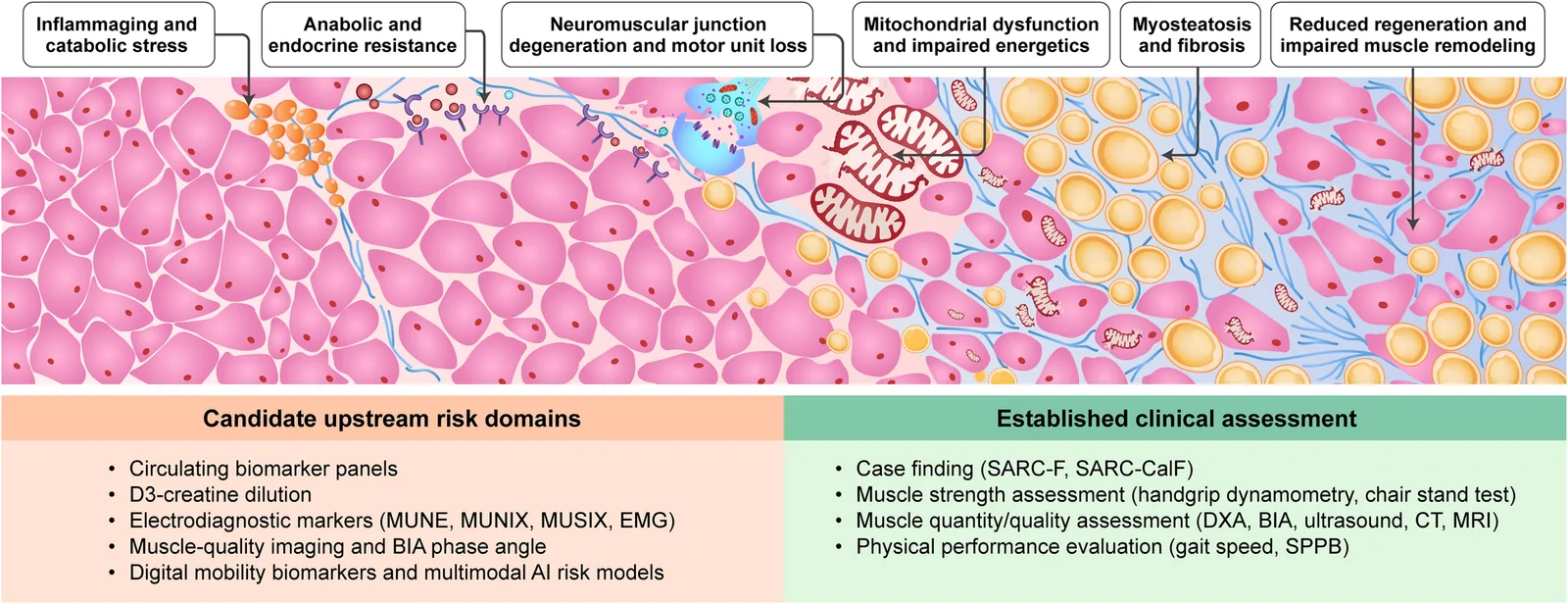
This conceptual composite depicts six labeled mechanisms, not a single individual, specimen, obligatory coexistence, or disease sequence. Structures are not to scale; arrows identify features rather than causal or temporal transitions. Tissue appearance cannot establish regenerative capacity. Five candidate measurement families accompany established assessments without implying sequential testing, diagnostic equivalence, or earlier abnormality. Imaging and BIA phase angle represent distinct constructs; clinical modalities are not interchangeable confirmatory tests. AI models, grouped with digital mobility for presentation, represent cross-domain analytical approaches, not another measurement family or models validated here. Proposed predictive and clinical applications require prospective validation (Figure 2). AI, artificial intelligence; BIA, bioelectrical impedance analysis; CT, computed tomography; D3-creatine, deuterated creatine; DXA, dual-energy X-ray absorptiometry; EMG, electromyography; MRI, magnetic resonance imaging; MUNE, motor unit number estimation; MUNIX, motor unit number index; MUSIX, motor unit size index; SPPB, Short Physical Performance Battery. SARC-F assesses strength, assistance with walking, rising from a chair, climbing stairs, and falls; SARC-CalF incorporates calf circumference.

**Figure 2:**
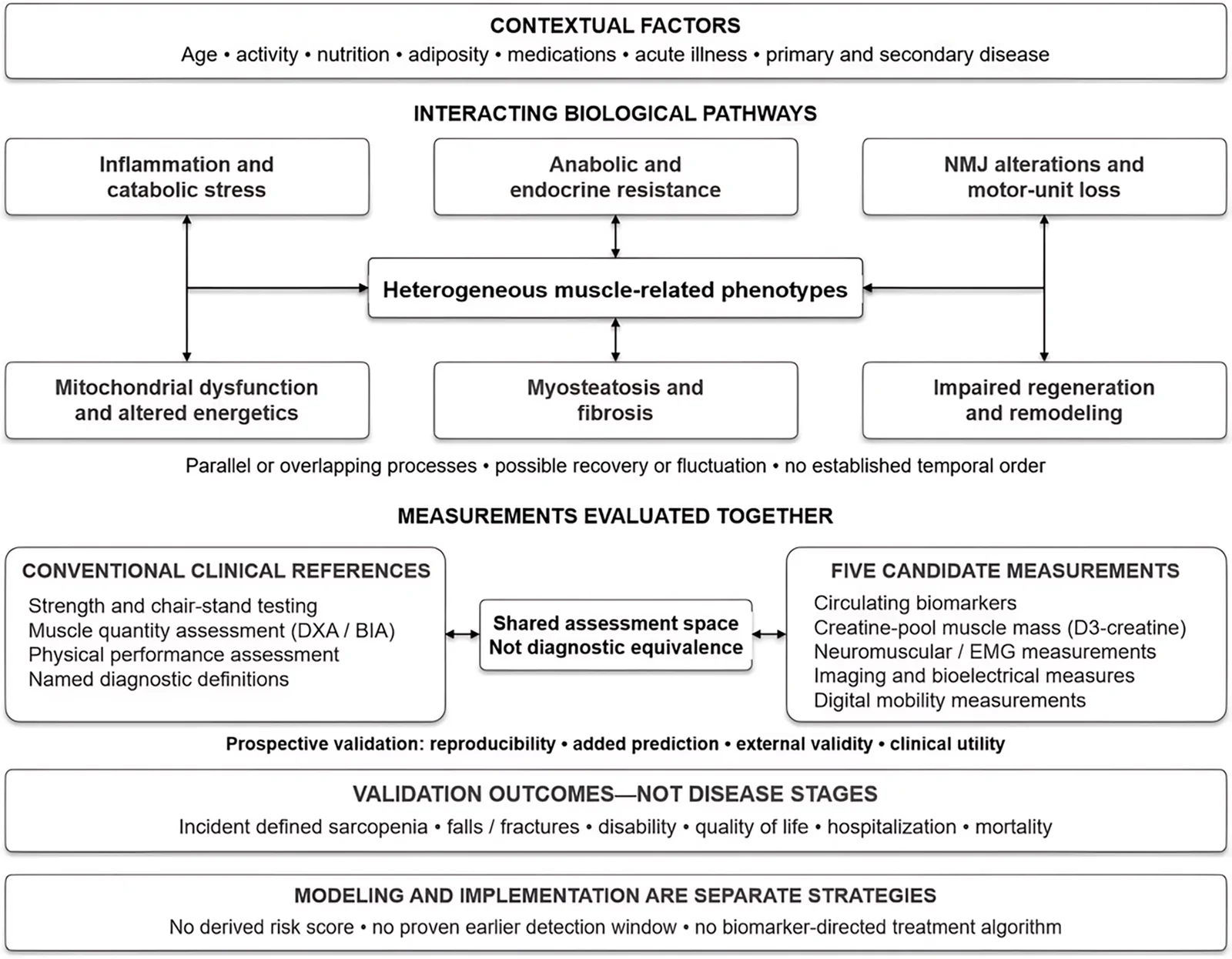
Contextual factors, including coexisting age- and disease-related contributors, frame overlapping pathways and heterogeneous phenotypes without fixed temporal ordering. Fluctuation or recovery may occur; arrows indicate conceptual, not demonstrated causal, relationships. Five candidate measurement families and conventional references are evaluated jointly, without diagnostic equivalence, mandatory combined testing, or equal evidence. Validation requires reproducibility, incremental prediction beyond clinical measures, external validity, and clinical utility. Operationally defined incident sarcopenia and other clinical outcomes are validation endpoints, not stages (8, 69–71). Tables 2–5 detail constructs, evidence, and research questions. Modeling and implementation are separate strategies. The framework does not derive a risk score, establish an earlier-detection window, or validate biomarker-directed treatment selection. BIA, bioelectrical impedance analysis; DXA, dual-energy x-ray absorptiometry; EMG, electromyography; NMJ, neuromuscular junction.

## 6 Candidate domains: demonstrated evidence and proposed applications

For each domain, the relevant questions are different: does a measurement distinguish current groups, predict incident sarcopenia, predict another adverse outcome, add information beyond conventional tools, respond to change, or identify differential treatment benefit? An affirmative answer to one question does not answer the others. Tables 3, 4 separates these evidence categories and distinguishes demonstrated measurement uses from unvalidated applications (18, 19). The five measurement families group methods for discussion; they do not imply equal evidence, interchangeable constructs, or a one-to-one mapping to the six mechanisms in Figure 1. Tables 3, 4 further separates routine blood indices from CAF and distinguishes CT/MRI, ultrasound, and phase angle within the imaging/bioelectrical family.

**Table 3:** Evidence and clinical readiness of candidate domains: study design and predictive evidence.

Domain/key sources	Predominant evidence	Current-status association/accuracy	Longitudinal evidence and endpoint	External/incremental validation
Routine blood indices/panels (49–51)	Diagnostic-accuracy meta-analyses; cross-sectional panels.	Variable accuracy; no studied blood marker optimal in the Lian review (32 studies; 23,840 participants).	Incident-sarcopenia prediction is not established by the cited diagnostic-accuracy evidence.	No validated sequential laboratory pathway; added value over simple clinical tools needs testing.
CAF (12, 52–55)	Observational studies; meta-analysis; exploratory intervention and longitudinal reports.	Association in some cohorts; aging-related changes can lack sarcopenia specificity.	110-kDa CAF: imprecise incident low-mass association in older women; no significant low-strength association. Not a validated composite diagnosis.	Assay-specific findings and meta-analysis are not external validation of a calibrated risk model.
D3-creatine (56, 57)	Measurement studies and observational prognostic cohorts, summarized in reviews.	Estimates total-body muscle mass; not direct force or contractile protein.	Associations with functional/adverse outcomes in selected cohorts; distinct from incident sarcopenia.	Incremental value depends on comparator/outcome; no validated decision-impact pathway here.
Electrodiagnostics (12, 20, 43, 58)	Human cross-sectional/mechanistic studies; systematic review; technical studies..	Age-related motor-unit/NMJ abnormalities; limited specificity in Sarto et al.	Cross-sectional motor-unit enlargement patterns do not establish transition prediction.	No harmonized, externally validated upstream sarcopenia prediction rule in the selected evidence.
CT/MRI composition (9, 10, 39, 61, 62)	Observational imaging and prognostic studies; technical and disease-specific reviews.	Composition and clinical-phenotype associations; construct- and protocol-dependent.	Mobility/adverse-outcome associations; endpoints must not be relabeled incident sarcopenia.	Transportability depends on population and protocol; decision benefit is not established.
Ultrasound (59, 60)	Measurement, cross-sectional and cutoff literature summarized in narrative reviews.	Size/echo-intensity associations and heterogeneous reference values.	No universal pre-threshold trajectory or incident-risk cutoff established by these reviews.	Device/site/operator differences constrain generalization; incremental prediction needs testing.
Phase angle (38)	Systematic review/meta-analysis: 17 studies; 2,710 participants.	Accuracy subset: 2 studies/390 participants for low muscle radiodensity, not incident sarcopenia.	Prospective incident-sarcopenia prediction is not established by the cited synthesis.	All studies had higher risk of bias; low/very-low certainty; no validated preclinical threshold.
Digital mobility measurements (63–65)	Predominantly cross-sectional or internally evaluated classification studies.	Smart-insole study: 83 participants, 23 with sarcopenia; current-status comparison.	No demonstrated within-person pre-threshold detection or incident prediction in the cited studies.	Internal splits ≠ external validation; calibration, added value and net benefit need prospective testing.

Evidence statements refer to the sources mapped in this review, not an exhaustive claim that no other evidence exists. “Meta-analysis available” describes study design, not high certainty. Cohorts summarized in different reviews may overlap. Clinical-readiness categories are descriptive, not formal GRADE ratings.

**Table 4:** Evidence and clinical readiness of candidate domains: demonstrated use, proposed use, treatment-response evidence, and readiness.

Domain	Demonstrated measurement use	Proposed future application	Responsiveness/differential treatment benefit	Confounders/current readiness
Routine blood indices/panels	Laboratory measurement; concurrent association/accuracy assessment.	Low-cost predictors in prospectively developed models.	No marker-guided differential-benefit rule established in the selected evidence.	Kidney function, diet, illness and fluid shifts; exploratory adjunct variables, not a screening sequence.
CAF	Assay-specific biological characterization and observational associations.	Prognostic enrichment or repeated biological measurement.	Exploratory CAF change reported after intervention; baseline CAF-guided allocation of superior treatment not demonstrated.	Assay/fragment, kidney/neurological comorbidity, age; research marker, not a validated treatment selector.
D3-creatine	Research estimate of total-body muscle mass.	Incremental prognosis and evaluation of discordant phenotypes.	Can be a trial measurement; no validated result-based treatment assignment demonstrated here.	Sampling and conversion assumptions; specialist research measurement, not routine adjudication.
Electrodiagnostics	Physiological characterization; clinically indicated differential neurological assessment.	Test prediction beyond aging and comorbid neurological disease.	Responsiveness needs technique-specific testing; no MUNIX/MUSIX-guided differential exercise benefit established.	Neuropathy, radiculopathy, diabetes, method and muscle; specialist physiological research.
CT/MRI composition	Anatomical/compositional assessment; opportunistic measurement on existing images.	Added prognostic information and actionable assessment pathways.	Composition change does not prove that baseline imaging selects a superior intervention.	Acquisition, contrast, segmentation, body size; opportunistic research, not an endorsed new screening algorithm.
Ultrasound	Bedside/regional measurements under specified protocols.	Repeatable regional trajectories linked to meaningful outcomes.	Change must exceed measurement error; differential treatment benefit not established.	Operator, settings, pressure and depth; feasible measurement, unvalidated upstream decision rule.
Phase angle	Bioelectrical measurement; associations with body/tissue characteristics.	Longitudinal predictor or monitoring candidate with standardized conditions.	Change can reflect hydration/illness; does not validate phase-angle-directed treatment intensity.	Device, edema, fluid distribution and nutrition; exploratory upstream marker, low/very-low certainty in cited review.
Digital mobility measurements	Feature extraction and current-status classification in studied settings.	Home monitoring, calibrated prospective prediction and tested decision support.	Reliable change, intervention responsiveness and differential-benefit prediction are separate, unproven applications here.	Device, activity/task, missingness and comorbidity; feasibility-stage tools for this proposed use.

No biomarker-guided treatment-selection strategy is established by the evidence presented in this review. Routine clinical availability of an assay or device is not equivalent to readiness for the proposed upstream use. A prognostic association, responsive measurement and treatment-effect modifier are different findings. CAF, C-terminal agrin fragment; NMJ, neuromuscular junction; AI, artificial intelligence. The five measurement families are disaggregated by method in this table. AI-based analysis may be applied across families and is not a separate measurement domain.

### 6.1 Circulating biomarkers

Circulating markers may characterize inflammation, endocrine status, tissue turnover, or neuromuscular processes, but most are not specific to sarcopenia (18, 19, 48). Lian et al. synthesized 32 studies involving 23,840 participants and concluded that none of the evaluated blood biomarkers was optimal for screening or diagnosis (49). Creatinine/cystatin C-based indices show variable diagnostic accuracy across populations and operational definitions (50). They should not be presented as validated stand-alone tests or as an established first step in a sequential screening pathway.

Creatinine reflects muscle production as well as diet and renal handling; cystatin C is also affected by renal function and non-muscle factors. Acute illness, changing kidney function, edema, and fluid shifts can alter concentrations and weaken interpretation of their ratio (49, 50). Routine laboratory indices are therefore candidate low-cost variables for prospective prediction studies, especially when already available for clinical reasons. Their interpretation requires relevant illness and kidney-function information, and does not justify additional testing solely to assign a preclinical sarcopenia label.

IL-6, CRP, TNF-alpha, and GDF-15 may reflect inflammatory or catabolic burden; IGF-1, DHEA-S, testosterone, myostatin, irisin, P3NP, and FABP3 represent other endocrine or turnover-related processes (18, 19, 48, 51). A panel may improve biological description, but combining nonspecific variables does not establish a validated subtype or prediction model. Assay reproducibility, added prognostic information, and calibration require direct evaluation.

CAF has observational and meta-analytic support for association with sarcopenic phenotypes (52–54). Nevertheless, assay differences, comorbidity, and inconsistent associations with strength limit interpretation. The Sarto study provides an important counterpoint because age-related abnormalities did not consistently distinguish sarcopenic from nonsarcopenic older adults (12). Drey et al. assessed CAF in 69 prefrail adults in an intervention study and reported changes after vitamin D supplementation and exercise (53). This is exploratory evidence of biomarker responsiveness, not proof that baseline CAF identifies which intervention provides greater functional benefit. Incident-sarcopenia prediction, external validation, and treatment-selection utility remain separate requirements.

Longitudinal CAF evidence is not entirely absent. In older women, Kamiya et al. evaluated the 110-kDa fragment against later low muscle mass and strength (55). The adjusted association with incident low mass was imprecise and did not meet the conventional 0.05 significance threshold (odds ratio 2.22; 95% confidence interval 0.97–5.06); incident low strength was not significantly associated. This assay-specific, component-level result does not establish prediction of a composite sarcopenia diagnosis or a validated treatment rule.

### 6.2 D3-creatine dilution

D3-creatine dilution estimates the total-body creatine pool from oral labeled creatine dosing and urinary enrichment, and converts it to an estimate of skeletal muscle mass (56, 57). It is more directly linked to muscle tissue than dual-energy x-ray absorptiometry (DXA)-derived lean soft tissue, but still depends on measurement and conversion assumptions. It does not directly measure contractile protein content, force production, or muscle-specific strength.

The literature summarized in the cited reviews supports associations between D3-creatine-estimated muscle mass and functional or adverse clinical outcomes in selected cohorts (56, 57). Such prognostic associations and use as a research measurement are distinct from showing that a result resolves a clinically discordant case, identifies a treatment, or improves management. None of those clinical decision roles is established by the evidence presented here.

Implementation requires attention to dosing, sampling, laboratory procedures, interpretation in relevant disease settings, and population reference data (56, 57). The appropriate position in this framework is a specialist research measurement of total-body muscle mass. Incremental prediction beyond strength and conventional body-composition measures, and clinical utility of acting on the result, require prospective testing.

### 6.3 Electrodiagnostic and neuromuscular measurements

Electrodiagnostic measurements offer mechanistically detailed information but are not necessarily specific to sarcopenia. Similar findings can occur during neuromuscular aging and with neuropathy, radiculopathy, motor-neuron disorders, diabetes, and myopathy (11, 12, 20). Their principal current contribution to the proposed framework is physiological characterization, with diagnostic evaluation of suspected neurological disease performed according to clinical indications.

Motor unit number estimation (MUNE) provides an estimate, not a direct count, of functioning motor units. The motor unit number index (MUNIX) is an index derived from the compound muscle action potential and voluntary surface interference patterns; it is not an absolute motor-unit count. The motor unit size index (MUSIX) is calculated from compound muscle action potential amplitude divided by MUNIX and is a related size index, not a direct anatomical measurement of motor-unit territory (58). These measures should not be conflated with motor-unit potential size obtained by other electromyographic methods.

Piasecki et al. associated failure of compensatory motor-unit enlargement with sarcopenic status in older men (43). That cross-sectional observation concerns motor-unit remodeling and does not validate failure of MUSIX enlargement as a marker of transition to sarcopenia. Longitudinal studies using prespecified techniques are needed to determine whether a change in any such index precedes, predicts, or merely accompanies functional decline.

Needle, near-fiber, and single-fiber approaches assess different aspects of denervation, reinnervation, potential morphology, and transmission variability (12, 20). Invasiveness, operator dependence, and comorbid neurological disorders constrain general screening use. Surface electromyography (sEMG) can characterize task-dependent activation and fatigability, but conventional sEMG features are not direct measurements of junctional transmission or motor-unit number. Electrode geometry, adipose tissue, contraction level, normalization, filtering, and muscle selection affect interpretation. Disease-specific thresholds, reproducibility, and prospective added value remain insufficiently established for routine upstream screening (20).

### 6.4 Imaging composition and bioelectrical phase angle

CT, MRI, and ultrasound describe complementary structural or compositional properties, not interchangeable forms of muscle quality (39, 59, 60). CT attenuation and MRI fat fraction can characterize adipose infiltration, whereas ultrasound provides regional size, architecture, and echo intensity. Echo intensity can reflect more than one tissue property and is sensitive to acquisition settings, depth, probe pressure, and analysis methods (39, 59, 60).

Longitudinal observational studies relate muscle fat infiltration or related compositional measures to mobility outcomes (9, 10). Automated CT analysis offers an opportunity to examine existing clinical images, and prognostic associations have been reported (61). However, disease-specific evidence, including pancreatic cancer, should not be generalized without qualification to primary age-related sarcopenia (62). Imaging solely to assign an investigational risk domain is not advocated. Protocol, anatomical level, contrast/acquisition conditions, segmentation, and body-size adjustment must be defined for each intended use.

Phase angle reflects the relationship between resistance and reactance and is influenced by body-cell properties and fluid distribution. It is not a direct measure of muscle contractility or a tissue-specific assay (38). The cited systematic review included 17 studies and 2,710 participants; all included studies had higher risk of bias, and certainty ranged from low to very low. Pooled classification accuracy was based on only two studies with 390 participants and concerned low skeletal muscle radiodensity, not prospective incident sarcopenia (38).

Accordingly, reduced phase angle is not a defining feature of a preclinical stage. Validated thresholds for that purpose are lacking, and device, protocol, hydration, edema, acute illness, and nutritional status require consideration (38). Monitoring a change is also different from knowing whether that change exceeds measurement variability or is clinically meaningful. Prospective prediction and decision benefit must be demonstrated before a phase-angle-guided screening or intervention rule is adopted.

### 6.5 Digital mobility measurements and multimodal prediction

Digital measurements can quantify gait, plantar pressure, and movement characteristics, but the cited studies predominantly address current-status classification or technical feasibility (63–65). The smart-insole study by Kim et al. compared 83 participants, including 23 with sarcopenia, cross-sectionally (63). It did not establish prediction of incident sarcopenia or detect within-person deterioration before clinical thresholds were crossed.

Sensor fusion and skeleton-based models demonstrate candidate feature extraction and classification approaches (64, 65). Internal train/test evaluation is not equivalent to external validation, and laboratory task measurements are not automatically validated for unsupervised daily-life monitoring. These studies are included to illustrate feasibility and evidence gaps, not because they establish an earlier detection window. Repeated home measurements could be tested for monitoring, but sensitivity to meaningful change, adherence, missingness, and transfer across devices and populations remain research questions.

Multimodal prediction, including artificial intelligence (AI)-based modeling, is an analytical strategy rather than an additional biological or measurement domain. Its grouping with digital mobility measurements in Figure 1 is for presentation only; such models may integrate variables across all five measurement families. Figure 2 explicitly distinguishes modeling and implementation from the measurement families. No combined model is derived or recommended in this review. A future model would need a specified population, endpoint, time horizon, measurement schedule, and handling of missing data. It should be compared with inexpensive clinical predictors and assessed for calibration, external validity, and net benefit. TRIPOD + AI and PROBAST + AI provide relevant reporting and appraisal standards; compliance with reporting guidance alone does not establish clinical utility (66, 67).

### 6.6 Clinical assessment and research settings

Community and primary-care assessment should continue to use appropriate conventional case finding, strength and performance assessment, nutritional evaluation, and clinical assessment of reversible contributors (6, 7, 13, 68). These measures can support exercise and nutritional care without requiring biomarker-defined categories. Routine laboratory results obtained for other indications may provide context, but the review does not propose a new laboratory screening sequence.

Specialist or research settings may add detailed physiological, imaging, or biochemical measurements when the scientific question or differential diagnosis justifies them. This is a distinction in resources and assessment purpose, not a claim of greater sarcopenia specificity. Tiered assessment is one possible implementation strategy, not an additional measurement domain or a validated sequence of tests. Figure 2 places implementation outside the measurement families. Whether such a pathway is efficient or improves patient outcomes requires an implementation study.

## 7 Proposed nonsequential risk domains and validation questions

Figure 2 presents the author-proposed, nonsequential research framework, whereas Figure 1 serves as its illustrative biological overview. The framework distinguishes contextual factors, candidate measurement families, conventional clinical reference phenotypes, and separate validation outcomes. It was developed to organize research questions and is not attributed to GLIS, AWGS, or another consensus group. Numbered stages are not proposed because no validated boundaries, ordering, transition probabilities, or treatment implications support them.

Contextual factors include age, activity, nutritional status, adiposity, comorbidity, medication exposure, and recent illness. Age alone does not assign a risk category. Studies should evaluate whether particular factors or trajectories add useful information, rather than presume that all older adults occupy a discriminatory risk state. If an absolute-risk threshold is proposed, it must be derived and validated for a specified outcome and decision.

Circulating, creatine-pool, neuromuscular, imaging/bioelectrical, and digital domains may be abnormal concurrently or independently. They are nonexclusive and nonsequential. A participant may show neuromuscular abnormalities with preserved strength, low strength without low mass, or compositional abnormalities with a different functional profile. Apparent improvement may reflect recovery, treatment, resolution of illness, or measurement variation and must be interpreted accordingly.

Conventional diagnoses and clinical phenotypes remain reference descriptors, not successive levels in the proposed framework. The shared assessment space in Figure 2 means that conventional and candidate measurements can be evaluated together in the same participants; it does not imply diagnostic equivalence. Incident sarcopenia is a definition-based validation endpoint. Falls, fractures, disability, hospitalization, quality of life, and mortality are separate clinical outcomes with multifactorial determinants (8, 69–71). A fall or fracture is not evidence that a person traversed a sarcopenia stage sequence. Table 5 translates the framework into validation questions rather than intervention assignments.

**Table 5:** Nonsequential research domains and corresponding validation questions.

Framework element	Research description—not an entry rule	Required validation question	Appropriate study/safeguard
Contextual risk factors	Age, activity, nutrition, adiposity, illness, comorbidity and medication exposure.	Which variables or trajectories add useful risk information?	Cohort model with a specified endpoint/horizon; age alone does not create a risk category.
Circulating domain	Inflammatory, endocrine, turnover and NMJ-related assay results.	Do reproducible values add prediction beyond routine clinical measures?	Assay standardization; renal/illness adjustment; external validation; no preclinical diagnosis from a single abnormal test.
Creatine-pool domain	D3-creatine-derived total-body muscle mass.	Does this estimate improve prediction or decisions beyond alternative mass measures and strength?	Head-to-head prospective comparisons, followed by decision-impact evaluation.
Neuromuscular domain	Motor-unit estimates/indices, potential features and transmission variability.	Does a feature distinguish sarcopenia risk from aging or neurological comorbidity?	Standardized longitudinal physiology; prespecified differential-diagnosis and confounder assessment.
Imaging/bioelectrical domains	Distinct radiodensity, fat-fraction, echogenicity and electrical measurements.	Which construct predicts which outcome, and what change exceeds noise?	Separate construct validation; repeated measures; protocol/hydration control; no assumed common cutoff.
Digital mobility domain	Task or daily-life movement measurements.	Do reliable trajectories predict incident outcomes beyond clinic testing?	Prospective external validation across devices/settings; prespecified missing-data handling.
Conventional clinical reference phenotypes	Strength, quantity and performance categories under named definitions.	How does apparent marker performance vary with the reference definition?	Sensitivity analyses across operational criteria; no mapping to successive proposed stages.
Prospective validation outcomes	Incident sarcopenia under specified diagnostic criteria; falls, fractures, disability, quality of life, hospitalization and mortality.	Does information improve decisions or outcomes, not only association?	Endpoint-specific prognostic studies and decision-impact trials; outcomes remain outside the biological domains.

Elements may coexist, change independently or improve. No mutual exclusivity, fixed sequence, validated risk score or treatment allocation is implied. Testing treatment-effect modification requires a separate randomized design; no intervention is assigned by this table.

## 8 Discussion: a validation agenda

### 8.1 Longitudinal and comparative validation

Prospective cohorts should enroll participants characterized by conventional criteria and obtain repeated candidate measurements. Incident sarcopenia must be distinguished from worsening strength, mobility limitation, falls, and other outcomes. Prespecified analyses should examine multiple operational definitions and account for differences in classification (27–30). Demonstrating a cross-sectional association or an association with mortality does not by itself establish prediction of incident sarcopenia.

### 8.2 Measurement and model validity

Analytical reproducibility, biological interpretation, clinical prediction, and decision utility should be evaluated separately (18, 19). Repeated assessments require estimates of within-person biological variation, test–retest reliability, and measurement error. Hydration, recent exercise, illness, medication changes, device calibration, and operator procedures should be recorded as appropriate. Minimal detectable change concerns change beyond measurement noise; clinically meaningful change requires an external functional or patient-centered anchor. Neither can be inferred from a statistically significant group difference.

For prediction models, analytical and clinical validation must also address missing data, overfitting, calibration, external validation, and performance across age, sex, body size, ethnicity, and disease groups (66, 67). Neuromuscular protocols require consistent muscle selection, electrode procedures, contraction targets, and interpretation of coexisting neuropathy or myopathy (12, 20, 58). Models should show incremental value over simple clinical predictors and should not use measurements that effectively reproduce the reference definition without acknowledging this limitation.

### 8.3 Prognostic versus predictive enrichment

Prognostic association links a marker to an outcome. Prognostic enrichment selects a higher-risk population, potentially increasing event rates. Predictive enrichment selects people expected to obtain greater benefit from a particular treatment; this requires evidence of treatment-effect modification, preferably a prespecified marker-by-treatment interaction in a randomized study. An association with outcome, a within-group biomarker change, or statistical significance in one subgroup but not another is insufficient to establish differential benefit.

The LIFE and SPRINTT trials, exercise/nutrition trials, and related syntheses support intervention in the populations actually studied (14, 72–74). Studies of variation in strength response also motivate individualized clinical assessment (15, 75). They do not validate selection of a specific exercise or nutritional prescription by CAF, MUNIX, phase angle, myosteatosis, or any domain combination. Exploratory biomarker responsiveness, such as CAF change, should be distinguished from improved functional benefit under biomarker-guided care (53). Current evidence supports biological characterization and hypothesis generation, not the proposed use of these markers to allocate treatment.

### 8.4 Clinical utility and implementation

Before an investigational pathway is introduced, studies should define the action triggered by the result and compare that pathway with usual clinical assessment. Relevant endpoints include benefit, unnecessary testing, burden, cost, feasibility, and unequal access. Marker-guided care should be tested against standard prevention and rehabilitation, rather than against withholding appropriate care. The framework succeeds only if its validation questions lead to demonstrably better decisions or outcomes; technical novelty alone is insufficient.

## 9 Limitations

This narrative review uses purposive source selection by a single author and is vulnerable to selection and interpretive bias. The absence of a reproducible database search and historical screening counts prevents assessment of search completeness. The supplementary map describes cited sources, not all eligible evidence, and its source counts cannot be interpreted as independent cohorts or formal evidence grades. English-language full-text interpretation may introduce language bias. No new meta-analysis, formal risk-of-bias assessment, or prediction-model derivation was performed.

Much of the candidate-marker literature is cross-sectional or disease-specific. Mechanistic plausibility, measurement feasibility, and prognostic association are not equivalent to disease specificity or clinical utility. The domains overlap, lack validated classification boundaries, and may change with reversible illness and measurement conditions. As a conceptual composite, Figure 1 provides no evidence of within-person temporal ordering, coexistence of all depicted abnormalities, or histological diagnosis. Figure 2 organizes validation questions rather than demonstrated causal relationships. The framework therefore cannot assign an individual diagnostic stage, probability, or treatment. Its applicability and added value must be established prospectively.

## 10. Conclusions

Conventional sarcopenia assessment remains the basis for identifying clinically relevant muscle impairment and guiding appropriate care. The unresolved research question is whether additional biological or functional measurements provide reproducible and clinically useful information before, or alongside, those clinical phenotypes.

This review contributes an evidence-to-validation framework rather than a staging scheme or a validated multimodal model. Parallel candidate domains are distinguished from contextual risk factors, clinical reference phenotypes, and patient-centered outcomes. Longitudinal prediction, added value, reproducibility, and treatment-effect modification require different study designs. Until those requirements are met, investigational measurements should support research and clinically indicated differential assessment, not unvalidated preclinical labeling or biomarker-directed treatment allocation.
