# Supplementary material for: A research framework for evaluating upstream multimodal sarcopenia risk domains in older adults
Source: Front Med (Lausanne). 2026 Oct 14;13:1928432. doi: 10.3389/fmed.2026.1928432 (PMC13714515; doi:10.3389/fmed.2026.1928432)
Supplement: Supplementary file 1 [file Table1.docx]

**Supplementary Table 1. Purposive evidence-identification and narrative-selection framework**

**A. Domain codes and mapped cited-source counts**

This table documents purposive narrative identification and selection, not a reproducible systematic search or retrospectively applied eligibility criteria. D1–D9 are literature-identification domains; they are distinct from the five candidate measurement families. Counts are derived from the unchanged source–domain assignments in Supplementary Table 2.

| **Code** | **Literature-identification domain** | **Mapped sources, n** | **Reference numbers in Supplementary Table 2** |
| --- | --- | --- | --- |
| D1 | Diagnostic frameworks and related clinical phenotypes | 17 | 1–3, 6–7, 16–17, 22–31 |
| D2 | Clinical outcomes and prognostic evidence | 19 | 4–5, 8–10, 24, 28, 32–36, 55–57, 61, 69–71 |
| D3 | Biological mechanisms and muscle-related trajectories | 19 | 5, 9–12, 25, 40–47, 51–55 |
| D4 | Circulating and creatine-pool measurements | 12 | 18–19, 48–57 |
| D5 | Electrodiagnostic and neuromuscular measurements | 12 | 11–12, 20, 40–47, 58 |
| D6 | Imaging, body composition, and bioelectrical measures | 11 | 9–10, 37–39, 56–57, 59–62 |
| D7 | Digital mobility and AI-based measurement studies | 5 | 61–65 |
| D8 | Intervention evidence and measurement responsiveness | 11 | 13–15, 37, 44, 53, 68, 72–75 |
| D9 | Review, measurement, and prediction-model methodology | 6 | 18–19, 21, 58, 66–67 |

Counting convention: one distinct cited source is counted once in each assigned domain. There are 75 unique cited sources and 112 nonexclusive source–domain assignments. Domain totals therefore must not be summed to estimate unique studies, independent cohorts, database retrievals, or screened records. Counts do not measure evidence certainty.

Reference numbers correspond to the manuscript bibliography, and domain assignments are documented at the source level in Supplementary Table 2.

**B. Narrative-selection rationale and interpretation limits: D1–D3**

| **Code / domain** | **Representative concepts** | **Narrative priority and source rationale** | **Conceptual scope and interpretation limits** | **Role in the review** |
| --- | --- | --- | --- | --- |
| D1 Diagnostic frameworks and related clinical phenotypes (n = 17) | sarcopenia; EWGSOP/EWGSOP2; AWGS 2019/2025; SDOC; GLIS; KWGS; presarcopenia; possible/functional sarcopenia; dynapenia; frailty; muscle health | Consensus and conceptual articles, guidelines, definition comparisons, and phenotype-validation studies provide the clinical reference framework. Primary comparisons clarify differences in classification. | Related concepts are retained for comparison, not treated as equivalent diagnoses. Validation of a named clinical phenotype does not validate an investigational biological stage. | Distinguish established clinical descriptors from the author-proposed research framework. |
| D2 Clinical outcomes and prognostic evidence (n = 19) | strength; chair stand; gait speed; SPPB; mobility limitation; falls; fractures; disability; hospitalization; mortality; quality of life; incident sarcopenia | Longitudinal cohorts and outcome syntheses inform prognostic claims; cross-sectional studies remain useful for understanding determinants of performance and phenotype differences. | Current-status or cross-sectional findings are not excluded from the narrative, but cannot establish future risk. Distinguish incident sarcopenia from its individual components and other clinical outcomes. | Separate current clinical information, longitudinal associations, and endpoint-specific validation needs. |
| D3 Biological mechanisms and muscle-related trajectories (n = 19) | NMJ; motor-unit loss; denervation/reinnervation; anabolic resistance; inflammation; mitochondrial dysfunction; myosteatosis; fibrosis; regeneration; muscle trajectories | Human physiological, mechanistic, and trajectory studies are emphasized for human claims. Mechanistic reviews and selected preclinical studies provide biological context. | Between-group differences and animal findings do not establish a universal human sequence. Shared aging abnormalities, null findings, and conflicting results remain relevant. | Identify plausible interacting pathways while preserving limits on temporal and disease-specific inference. |

Narrative priority concerns emphasis, not formal exclusion. Null and discordant findings remain relevant. Limited external validation is reported as an evidence gap, not treated as an automatic eligibility failure. Counts and reference numbers are reported in part A.

**B. Narrative-selection rationale and interpretation limits: D4–D6**

| **Code / domain** | **Representative concepts** | **Narrative priority and source rationale** | **Conceptual scope and interpretation limits** | **Role in the review** |
| --- | --- | --- | --- | --- |
| D4 Circulating and creatine-pool measurements (n = 12) | IL-6; CRP; GDF-15; endocrine/turnover markers; creatinine/cystatin C; CAF; neurofilament light chain; D3-creatine dilution | Biomarker guidance, diagnostic-accuracy syntheses, observational studies, and measurement reviews characterize assays and creatine-pool-based mass estimation. Longitudinal and intervention reports are distinguished. | Nonspecificity is an interpretive limitation, not an automatic exclusion rule. Assay type, renal function, illness, and reference definition matter. D3-creatine estimates mass, not contractile function. | Separate biological description and measurement capability from unproven screening, prediction, monitoring, or treatment-selection uses. |
| D5 Electrodiagnostic and neuromuscular measurements (n = 12) | EMG; sEMG; near-fiber/single-fiber EMG; jitter; MUNE; MUNIX; MUSIX; motor-unit remodeling; transmission variability | Primary human physiological and technical studies support method-specific descriptions. Systematic and mechanistic reviews characterize the breadth and limitations of neuromuscular evidence. | Age effects and neurological comorbidity are not assumed to be sarcopenia-specific. Estimates, indices, and task-dependent activation are not interchangeable constructs; cross-sectional patterns do not establish transition prediction. | Define what each neuromuscular measure assesses and what longitudinal and differential-diagnostic validation is still required. |
| D6 Imaging, body composition, and bioelectrical measures (n = 11) | CT attenuation; MRI fat fraction; ultrasound thickness/architecture/echo intensity; BIA; phase angle; body composition; adiposity; sarcopenic obesity | Imaging/measurement studies, prognostic cohorts, relevant consensus statements, and modality-specific reviews characterize composition and bioelectrical constructs. Conventional mass comparisons provide context. | Size measures are retained when relevant to construct comparison. Distinguish imaging composition, bioelectrical properties, and mass. Device, protocol, hydration, and disease context constrain interpretation. | Compare distinct measurement constructs and their evidence, without implying a common quality cutoff or diagnostic interchangeability. |

Narrative priority concerns emphasis, not formal exclusion. Null and discordant findings remain relevant. Limited external validation is reported as an evidence gap, not treated as an automatic eligibility failure. Counts and reference numbers are reported in part A.

**B. Narrative-selection rationale and interpretation limits: D7–D9**

| **Code / domain** | **Representative concepts** | **Narrative priority and source rationale** | **Conceptual scope and interpretation limits** | **Role in the review** |
| --- | --- | --- | --- | --- |
| D7 Digital mobility and AI-based measurement studies (n = 5) | wearable sensors; smart insoles; gait/plantar pressure; sensor fusion; pose/skeleton analysis; machine learning; automated CT; digital mobility | Technical, cross-sectional, internally evaluated, and relevant imaging-model studies illustrate feature extraction, feasibility, and existing evidence gaps. | Absence of external validation is a limitation, not an automatic reason for removal. Internal evaluation is not external validation; laboratory feasibility does not establish home monitoring or prospective risk prediction. | Distinguish measured signals from modeling strategies and define the additional evidence required for longitudinal or clinical use. |
| D8 Intervention evidence and measurement responsiveness (n = 11) | exercise; protein/nutritional support; multicomponent intervention; rehabilitation; functional outcomes; repeated measurements; response heterogeneity; predictive enrichment | Trials and intervention syntheses inform efficacy in the populations studied. Guidelines and contextual or mechanistic sources inform interpretation; biomarker analyses inform exploratory measurement responsiveness. | Measurement change or response heterogeneity does not establish differential treatment benefit. No reviewed source is treated as validation of stage- or biomarker-directed treatment assignment. | Summarize intervention efficacy in studied populations and measurement responsiveness. Predictive enrichment remains a future research question requiring separate treatment-effect-modification evidence. |
| D9 Review, measurement, and prediction-model methodology (n = 6) | SANRA; biomarker/measurement methodology; MUNIX principles; TRIPOD+AI; PROBAST+AI; reproducibility; calibration; external validation; clinical utility | Review-appraisal, measurement, biomarker, and prediction-model guidance provide terminology and methodological standards appropriate to each claim. | Use of guidance does not mean that this review performed a formal appraisal, developed software as a research output, or validated a model. This domain is not limited to prediction modeling. | Define reporting and validation requirements; distinguish descriptive evidence mapping from formal analysis or model validation. |

Narrative priority concerns emphasis, not formal exclusion. Null and discordant findings remain relevant. Limited external validation is reported as an evidence gap, not treated as an automatic eligibility failure. Counts and reference numbers are reported in part A.
