# Supplementary material for: A research framework for evaluating upstream multimodal sarcopenia risk domains in older adults
Source: Front Med (Lausanne). 2026 Oct 14;13:1928432. doi: 10.3389/fmed.2026.1928432 (PMC13714515; doi:10.3389/fmed.2026.1928432)
Supplement: Supplementary file 2 [file Table2.docx]

**Supplementary Table 2. Source-level evidence map**

**Domain-code key and counting convention**

Unit of mapping: one cited source record, not one independent study or cohort. The 75 records comprise the 71-source initial bibliography plus four additions from the first revision. Each record is identified by its manuscript reference number and mapped to its narrative domain(s), evidence type, supported inference, and key interpretive limitation.

| **Code** | **Literature-identification domain** | **Mapped cited sources, n** |
| --- | --- | --- |
| D1 | Diagnostic frameworks and related clinical phenotypes | 17 |
| D2 | Clinical outcomes and prognostic evidence | 19 |
| D3 | Biological mechanisms and muscle-related trajectories | 19 |
| D4 | Circulating and creatine-pool measurements | 12 |
| D5 | Electrodiagnostic and neuromuscular measurements | 12 |
| D6 | Imaging, body composition, and bioelectrical measures | 11 |
| D7 | Digital mobility and AI-based measurement studies | 5 |
| D8 | Intervention evidence and measurement responsiveness | 11 |
| D9 | Review, measurement, and prediction-model methodology | 6 |

The same D1–D9 codes, domain names, and counts appear in Supplementary Table 1. A source is counted once per assigned domain; nonexclusive assignment gives 112 source–domain links across 75 unique sources. These counts are not search yield, screening counts, independent replications, or evidence grades.

Domain labels describe the narrative roles of sources, not a uniform design or endpoint within a domain. For example, D8 includes intervention trials, response analyses, guidelines, and contextual or mechanistic reports. It does not mean that every source tested efficacy or biomarker-by-treatment interaction. D9 includes narrative-review and measurement methodology as well as prediction-model guidance.

Full bibliographic details and DOIs remain in the manuscript References. The following nine table parts present the source-level evidence map. No additional literature search, formal risk-of-bias assessment, or prediction-model validation was undertaken to produce this descriptive map.

# Supplementary Table 2. Source-level evidence map (1/9)

| **Reference / source** | **Domains** | **Design / evidence type** | **What the source supports in this review** | **Inference not established / key limitation** |
| --- | --- | --- | --- | --- |
| 1. Rosenberg IH (1997) | D1 | Historical/conceptual article | Terminology and clinical origins of sarcopenia | No diagnostic or prognostic validation |
| 2. Baumgartner RN et al. (1998) | D1 | Population cross-sectional study | Mass-based operational phenotype and prevalence | No pre-threshold temporal sequence |
| 3. Cruz-Jentoft AJ et al. (2010) | D1 | Consensus statement | EWGSOP 2010 definition and presarcopenia | Not the proposed domain framework |
| 4. Newman AB et al. (2006) | D2 | Prospective observational cohort | Strength and mortality association | Association is not marker-guided care |
| 5. Goodpaster BH et al. (2006) | D2, D3 | Longitudinal observational cohort | Differing strength and mass trajectories | No universal mechanistic ordering |
| 6. Cruz-Jentoft AJ et al. (2019) | D1 | Consensus statement | EWGSOP2 probable, confirmed and severe categories | No validation of investigational stage boundaries |
| 7. Chen LK et al. (2020) | D1 | Consensus statement | AWGS 2019 criteria and possible sarcopenia | Clinical categories, not a biological stage sequence |
| 8. Beaudart C et al. (2025) | D2 | Outcome consensus report | Patient-relevant sarcopenia outcomes | Outcomes are not proposed stages |
| 9. Delmonico MJ et al. (2009) | D2, D3, D6 | Longitudinal observational cohort | Strength, composition and adipose-infiltration trajectories | No treatment allocation rule |

This descriptive map is not a systematic study inventory, a formal risk-of-bias assessment, or an evidence-certainty score. Domains are nonexclusive; source and participant overlap may occur. Full bibliographic details and DOIs appear in the manuscript.

# Supplementary Table 2. Source-level evidence map (2/9)

| **Reference / source** | **Domains** | **Design / evidence type** | **What the source supports in this review** | **Inference not established / key limitation** |
| --- | --- | --- | --- | --- |
| 10. Visser M et al. (2005) | D2, D3, D6 | Prospective observational cohort | Muscle composition and incident mobility limitation | Mobility limitation is not incident sarcopenia |
| 11. Arnold WD et al. (2023) | D3, D5 | Mechanistic narrative review | NMJ transmission hypotheses in aging | Mechanistic plausibility is not disease specificity |
| 12. Sarto F et al. (2024) | D3, D5 | Human cross-sectional mechanistic study | 130 participants; many abnormalities shared by older sarcopenic and nonsarcopenic groups | No prospective incidence; limited current specificity |
| 13. Dent E et al. (2018) | D8 | Clinical practice guideline | Conventional screening and exercise/nutrition care | No CAF-, MUNIX- or phase-angle-directed care |
| 14. Bernabei R et al. (2022) | D8 | Randomized multicomponent trial (SPRINTT) | Mobility-disability prevention in the enrolled frail population | Not a trial of biomarker-guided treatment selection |
| 15. Churchward-Venne TA et al. (2015) | D8 | Exercise intervention/response analysis | Variation in response to resistance training | Does not establish biomarker-by-treatment interaction |
| 16. Kirk B et al. (2024) | D1 | Delphi consensus | Components, reversibility; severity levels did not achieve the acceptance threshold | Does not endorse six stages or all quality measures as equivalent |
| 17. Chen LK et al. (2025) | D1 | Consensus update | Life-course muscle health; 50–64-year assessment; mass and strength diagnosis | Not the proposed evidence-to-validation framework |
| 18. Cesari M et al. (2012) | D4, D9 | Biomarker consensus/methodological guidance | Biomarker roles and validation in trials | Not validation of a new panel or clinical decision rule |

This descriptive map is not a systematic study inventory, a formal risk-of-bias assessment, or an evidence-certainty score. Domains are nonexclusive; source and participant overlap may occur. Full bibliographic details and DOIs appear in the manuscript.

# Supplementary Table 2. Source-level evidence map (3/9)

| **Reference / source** | **Domains** | **Design / evidence type** | **What the source supports in this review** | **Inference not established / key limitation** |
| --- | --- | --- | --- | --- |
| 19. Ladang A et al. (2023) | D4, D9 | Biochemical-marker consensus | Candidate biological domains and trial measurement | No validated mechanism-specific treatment assignment |
| 20. Cui C et al. (2026) | D5 | Systematic review of EMG evidence | Motor-unit/NMJ alterations across heterogeneous aging studies | Study designs do not establish a universal temporal sequence |
| 21. Baethge C et al. (2019) | D9 | Narrative-review appraisal methodology | Transparency and scientific reasoning for narrative reviews | Not a substitute for a reproducible search or formal risk-of-bias assessment |
| 22. Bhasin S et al. (2020) | D1 | Consortium position statement | Outcome-oriented strength/performance definition | Reference phenotype differs from mass-based criteria |
| 23. Baek JY et al. (2023) | D1 | Regional expert consensus | KWGS screening and functional sarcopenia | Not validation of proposed upstream domains |
| 24. Ji S et al. (2024) | D1, D2 | Observational phenotype-validation study | Comparison of KWGS clinical phenotypes | Clinical phenotype validation is not preclinical prediction |
| 25. Clark BC et al. (2008) | D1, D3 | Conceptual review | Dynapenia distinguished from muscle-mass loss | No new diagnostic staging criteria |
| 26. Rockwood K et al. (2005) | D1 | Clinical frailty-scale validation study | Broader health/functional vulnerability beyond skeletal muscle | Frailty is not interchangeable with muscle-specific domain assessment |
| 27. Stuck AK et al. (2023) | D1 | Cross-sectional definition comparison | Variation in prevalence under 12 definitions | Agreement does not establish a biological reference standard |

This descriptive map is not a systematic study inventory, a formal risk-of-bias assessment, or an evidence-certainty score. Domains are nonexclusive; source and participant overlap may occur. Full bibliographic details and DOIs appear in the manuscript.

# Supplementary Table 2. Source-level evidence map (4/9)

| **Reference / source** | **Domains** | **Design / evidence type** | **What the source supports in this review** | **Inference not established / key limitation** |
| --- | --- | --- | --- | --- |
| 28. Westbury LD et al. (2023) | D1, D2 | Population cohort comparison with mortality follow-up | Definition agreement and mortality associations | Mortality association does not establish incident-sarcopenia prediction |
| 29. Van Ancum JM et al. (2020) | D1 | Observational definition comparison | Effect of switching to EWGSOP2 | No validation of investigational marker-guided care |
| 30. Wallengren O et al. (2021) | D1 | Two-cohort definition comparison | Classification under EWGSOP 2010 and 2019 | Different labels do not prove temporal transitions |
| 31. Petermann-Rocha F et al. (2022) | D1 | Systematic review/meta-analysis of prevalence | Heterogeneity in prevalence across definitions | Prevalence synthesis is not prognostic validation |
| 32. Guralnik JM et al. (1994) | D2 | Observational prognostic study | SPPB and subsequent important geriatric outcomes | Integrated performance is not muscle-specific pathology |
| 33. Studenski S et al. (2011) | D2 | Pooled cohort prognostic analysis | Usual gait speed and survival | No preclinical marker or treatment-selection validation |
| 34. Rosso AL et al. (2015) | D2 | Longitudinal observational study | Multisystem impairments and gait-speed change | Gait change is not specific to sarcopenia |
| 35. Tiedemann A et al. (2005) | D2 | Observational predictor study | Physiological/psychological correlates of walking speed | No marker-guided intervention comparison |
| 36. Lord SR et al. (2002) | D2 | Cross-sectional performance study | Non-strength determinants of sit-to-stand performance | Functional testing does not isolate a single mechanism |

This descriptive map is not a systematic study inventory, a formal risk-of-bias assessment, or an evidence-certainty score. Domains are nonexclusive; source and participant overlap may occur. Full bibliographic details and DOIs appear in the manuscript.

# Supplementary Table 2. Source-level evidence map (5/9)

| **Reference / source** | **Domains** | **Design / evidence type** | **What the source supports in this review** | **Inference not established / key limitation** |
| --- | --- | --- | --- | --- |
| 37. Donini LM et al. (2022) | D6, D8 | ESPEN/EASO consensus statement | Sarcopenic obesity definition and contextual interpretation | Not automatic sarcopenia classification based on obesity alone |
| 38. da Costa Pereira JP et al. (2024) | D6 | Systematic review/meta-analysis | 17 studies, 2,710 participants; low/very-low certainty; accuracy subset 2 studies/390 for low radiodensity | Not prospective incident-sarcopenia validation; higher risk of bias |
| 39. Lavalle S et al. (2024) | D6 | Imaging/assessment narrative review | Different imaging and non-radiation measurement constructs | Not validation of interchangeability or a common quality cutoff |
| 40. Campbell MJ et al. (1973) | D3, D5 | Human cross-sectional physiological study | Age-associated muscle/motor-unit differences | Between-age differences do not establish within-person progression |
| 41. McNeil CJ et al. (2005) | D3, D5 | Human cross-sectional motor-unit study | MUNE differences in young, old and very old men | No longitudinal transition threshold |
| 42. Piasecki M et al. (2016) | D3, D5 | Physiological narrative review | Age-related motor-unit remodeling | No disease-specific screening rule |
| 43. Piasecki M et al. (2018) | D3, D5 | Human cross-sectional motor-unit study | Failure of compensatory motor-unit enlargement associated with sarcopenic status | Not a longitudinal MUSIX transition study |
| 44. Aagaard P et al. (2010) | D3, D5, D8 | Mechanistic/intervention narrative review | Neural contributions and strength-training rationale | No MUNIX-based allocation of exercise treatment |
| 45. Deschenes MR et al. (2010) | D3, D5 | Preclinical animal mechanistic study | NMJ remodeling preceding selected myofiber alterations | Animal ordering cannot be assumed in human sarcopenia |

This descriptive map is not a systematic study inventory, a formal risk-of-bias assessment, or an evidence-certainty score. Domains are nonexclusive; source and participant overlap may occur. Full bibliographic details and DOIs appear in the manuscript.

# Supplementary Table 2. Source-level evidence map (6/9)

| **Reference / source** | **Domains** | **Design / evidence type** | **What the source supports in this review** | **Inference not established / key limitation** |
| --- | --- | --- | --- | --- |
| 46. Gonzalez-Freire M et al. (2014) | D3, D5 | Mechanistic narrative review | Aging changes at the NMJ | No validated human preclinical staging scheme |
| 47. Ham DJ et al. (2018) | D3, D5 | Mechanistic narrative review | Causes and consequences of NMJ aging | No clinical prediction or intervention-selection validation |
| 48. El-Sebaie M et al. (2023) | D4 | Biomarker narrative review | Breadth and nonspecificity of candidate blood markers | Contextual review, not primary diagnostic validation |
| 49. Lian R et al. (2024) | D4 | Systematic review/meta-analysis of diagnostic accuracy | 32 studies; 23,840 participants; no evaluated blood marker optimal for screening/diagnosis | Prevalent-status accuracy does not establish prediction or utility |
| 50. Lin T et al. (2024) | D4 | Systematic review/meta-analysis of diagnostic accuracy | Definition-dependent performance of creatinine/cystatin C indices | No validated sequential laboratory screening pathway |
| 51. Qaisar R et al. (2021) | D3, D4 | Cross-sectional biomarker-panel study | Association/classification using a circulating panel | Not a prospectively validated incident-risk model |
| 52. Pratt J et al. (2021) | D3, D4 | Cross-sectional GenoFit study | CAF association in 300 adults aged 50–83 years | Early biomarker in the title does not prove longitudinal early detection |
| 53. Drey M et al. (2013) | D3, D4, D8 | Biomarker analysis within an intervention study | 69 prefrail participants; CAF responsiveness after intervention | Change does not establish baseline CAF prediction of differential benefit |
| 54. Fatima R et al. (2025) | D3, D4 | Systematic review/meta-analysis | Observational association of CAF with sarcopenia; meta-analysis available | Assay heterogeneity and observational design limit clinical readiness |

This descriptive map is not a systematic study inventory, a formal risk-of-bias assessment, or an evidence-certainty score. Domains are nonexclusive; source and participant overlap may occur. Full bibliographic details and DOIs appear in the manuscript.

# Supplementary Table 2. Source-level evidence map (7/9)

| **Reference / source** | **Domains** | **Design / evidence type** | **What the source supports in this review** | **Inference not established / key limitation** |
| --- | --- | --- | --- | --- |
| 55. Kamiya K et al. (2023) | D2, D3, D4 | Observational cohort with longitudinal follow-up | 110-kDa CAF in older women; incident low-mass OR 2.22 (95% CI 0.97–5.06); no significant low-strength association | Component-level, imprecise result; no composite diagnosis or treatment-selection validation |
| 56. Pagano AP et al. (2024) | D2, D4, D6 | Narrative review of D3-creatine studies | Muscle-mass measurement and selected prognostic associations | Not itself a clinical decision-impact study; may overlap reviewed cohorts |
| 57. Evans WJ et al. (2024) | D2, D4, D6 | D3-creatine methodological/narrative review | Creatine-pool-based mass estimation and aging-research applications | No validated discordant-case adjudication or treatment rule |
| 58. Nandedkar SD et al. (2010) | D5, D9 | Technical/methodological human study | MUNIX construction; MUSIX size-index interpretation | Index values are not direct anatomical motor-unit counts or territories |
| 59. Prell T et al. (2024) | D6 | Ultrasound narrative review | Bedside measurement, feasibility and limitations | No universal ultrasound threshold for preclinical risk |
| 60. Van den Broeck J et al. (2025) | D6 | Ultrasound cutoff narrative review | Population- and protocol-dependent ultrasound reference measures | Limited generalizability; not a validated universal early-detection rule |
| 61. Pickhardt PJ et al. (2022) | D2, D6, D7 | Retrospective imaging prognostic study | Automated L1/L3 CT measures and adverse-outcome associations | Not a randomized test of CT-guided care |
| 62. Gehin W et al. (2025) | D6, D7 | Disease-specific technical/clinical narrative review | AI CT applications in pancreatic cancer | Secondary-disease evidence is not automatically generalizable to primary sarcopenia |
| 63. Kim S et al. (2023) | D7 | Cross-sectional smart-insole study | 83 participants including 23 with sarcopenia; current-status differences | No within-person pre-threshold deterioration or incident prediction |

This descriptive map is not a systematic study inventory, a formal risk-of-bias assessment, or an evidence-certainty score. Domains are nonexclusive; source and participant overlap may occur. Full bibliographic details and DOIs appear in the manuscript.

# Supplementary Table 2. Source-level evidence map (8/9)

| **Reference / source** | **Domains** | **Design / evidence type** | **What the source supports in this review** | **Inference not established / key limitation** |
| --- | --- | --- | --- | --- |
| 64. Aznar-Gimeno R et al. (2024) | D7 | Cross-sectional sensor-fusion/model study | Candidate gait signals and current-status classification | Internal evaluation is not external prospective clinical validation |
| 65. Naseem MT et al. (2024) | D7 | Cross-sectional multimodal model study | Skeleton/foot-pressure features for current-status classification | No established incremental incident prediction or clinical utility |
| 66. Collins GS et al. (2024) | D9 | Prediction-model reporting guideline | TRIPOD+AI transparency and reporting requirements | Reporting compliance is not validation or clinical utility |
| 67. Moons KGM et al. (2025) | D9 | Prediction-model risk-of-bias/applicability methodology | PROBAST+AI appraisal requirements | This review did not formally score candidate models with the tool |
| 68. Xie WQ et al. (2020) | D8 | Narrative clinical review | Possible sarcopenia and early conventional care | No validation of biomarker-defined treatment timing |
| 69. Yeung SSY et al. (2019) | D2 | Systematic review/meta-analysis of observational outcomes | Sarcopenia associations with falls and fractures | Multifactorial outcomes do not imply a fixed stage sequence |
| 70. Xu J et al. (2022) | D2 | Systematic review/meta-analysis of mortality associations | Sarcopenia and mortality association | No marker-guided management validation |
| 71. Beaudart C et al. (2023) | D2 | Systematic review/meta-analysis of quality of life | Association with health-related quality of life | Not a preclinical stage or treatment-allocation endpoint by itself |
| 72. Pahor M et al. (2014) | D8 | Randomized activity trial (LIFE) | Physical activity and prevention of mobility disability | Not an upstream biomarker-allocation trial |

This descriptive map is not a systematic study inventory, a formal risk-of-bias assessment, or an evidence-certainty score. Domains are nonexclusive; source and participant overlap may occur. Full bibliographic details and DOIs appear in the manuscript.

# Supplementary Table 2. Source-level evidence map (9/9)

| **Reference / source** | **Domains** | **Design / evidence type** | **What the source supports in this review** | **Inference not established / key limitation** |
| --- | --- | --- | --- | --- |
| 73. Chang KV et al. (2021) | D8 | Randomized early-versus-delayed intervention trial | Exercise/nutrition timing in sarcopenic older adults | Not preclinical biomarker-directed treatment |
| 74. Whaikid P et al. (2024) | D8 | Systematic review/meta-analysis of interventions | Protein plus resistance-exercise effects in studied populations | Does not establish phenotype-specific benefit by candidate markers |
| 75. Clark LA et al. (2021) | D8 | Training-response study | Heterogeneity of strength response and contractile contributions | Response heterogeneity alone does not validate treatment-effect modification |

This descriptive map is not a systematic study inventory, a formal risk-of-bias assessment, or an evidence-certainty score. Domains are nonexclusive; source and participant overlap may occur. Full bibliographic details and DOIs appear in the manuscript.
